# Hyperspectral time series datasets of maize during the grain filling period

**DOI:** 10.1186/s13104-022-06029-9

**Published:** 2022-04-29

**Authors:** Valerie Craig, Hugh Earl, John Sulik, Elizabeth A. Lee

**Affiliations:** University of Guelph, Guelph, ON N1G 2W1 Canada

**Keywords:** Maize, *Zea mays* L, Hyperspectral, Remote sensing, Grain filling period, Physiological maturity, Development, Reflectance, Dual-channel unispec

## Abstract

**Objectives:**

Remotely sensed hyperspectral data are increasingly being used to assess crop development and growth throughout the growing season. Large datasets capturing key growth stages can be useful to researchers studying many physiological plant responses. A time series analysis of hyperspectral reflectance measurements taken during the grain filling period and published within a publicly accessible database are described herein. These datasets document the spectral reflectance pattern of the canopy within the visible and near-infrared portion of the electromagnetic spectrum during the late stages of the grain filling period as plants approach and reach physiological maturity.

**Data description:**

Included within the data repository are canopy-level hyperspectral datasets collected in 2017 and 2018. Data is included in its raw form, as well as with several manipulations to smooth and standardize the raw data. Data are released as comma separated value spreadsheets as well as Microsoft Excel open XLSX spreadsheets. These are accompanied by README text files which further describe the data and supplemental files that record hybrids used and plant phenology for each year of data collection.

## Objective

Characterization of plant development and growth throughout the plant’s lifecycle using hyperspectral remote sensing technologies is an attractive alternative to many traditional phenotyping approaches [1]. Time series analyses of crop growth is particularly useful for researchers to analyze the spectral changes occurring through different growth stages or critical phases of development [2]. Maize (*Zea mays* L.) has several key growth stages: vegetative growth, flowering, the grain-filling period, and physiological maturity (a.k.a., black layer) [3]. Many spectral reflectance-based phenotyping approaches are being examined for their utility in maize [3, 4]. In this paper we describe a set of spectral reflectance data collected from 16 single-cross maize hybrids during the later stages of growth for 2 years (2017 & 2018). This dataset specifically describes canopy reflectance in 3 nm increments from the visible through the near infrared portion of the electromagnetic spectrum starting at 3-weeks post silking and continuing until physiological maturity.

## Data description

Included within the data repository are several hyperspectral reflectance datasets and their manipulations. Files follow a naming structure of YEAR_DATA TYPE_MANIPULATION (Data Files 1–20, Table 1), where YEAR can be either 2017 or 2018, DATA TYPE is either the full dataset or the data averaged for every genotype on each sampling date across replications, and MANIPULATION is raw data, data that has been standardized by dividing reflectance at every wavelength by reflectance at 849.5 nm, data smoothed by calculating a rolling average every 9.9 nm, or data which has been standardized and smoothed. Missing values from the hyperspectral sensor and skipped scans are populated with a decimal and treated as missing data for all manipulations. In addition to these spectral reflectance data files, supplementary files are also present in the repository. Data collection methods and manipulations are described in the Data File 21 README file, and variable names and descriptions are included in the Data File 22 CODEBOOK file (Table 1). Information about the single-cross hybrids used in the study and their inbred parents are found in the Data File 23 GENETIC_MATERIALS datafile (Table 1). Date of flowering (i.e., appearance of the silks) and of physiological maturity (i.e., appearance of black layer) are found in Data File 24 and 25 PHENOLOGY datafiles for 2017 and 2018 (Table 1). A SUPPLEMENTARY_IMAGES file is also included (Data File 26), which contains images of the experimental setup, examples of black layer, and an example graph of spectral reflectance (Table 1). All files contained within the data repository and their file type can be found in Table 1.

**Table 1:** Dataverse repository file folder structure

Label	Name of data file / data set	File types (file extension)	Data Repository and Identifier (DOI or Accession Number)
Data File 1	2017_FULL_ALLDATA (2017 folder)	.xlsx	Scholars Portal Dataverse, https://doi.org/10.5683/SP2/1ZVWFV [5]
Data File 2	2017_FULL_RAW (2017 folder)	.csv	Scholars Portal Dataverse, https://doi.org/10.5683/SP2/1ZVWFV [5]
Data File 3	2017_FULL_STANDARDIZED (2017 folder)	.csv	Scholars Portal Dataverse, https://doi.org/10.5683/SP2/1ZVWFV [5]
Data File 4	2017_FULL_SMOOTHED (2017 folder)	.csv	Scholars Portal Dataverse, https://doi.org/10.5683/SP2/1ZVWFV [5]
Data File 5	2017_FULL_STANDARDIZED_SMOOTHED (2017 folder)	.csv	Scholars Portal Dataverse, https://doi.org/10.5683/SP2/1ZVWFV [5]
Data File 6	2017_AVERAGE_ALLDATA (2017 folder)	.xlsx	Scholars Portal Dataverse, https://doi.org/10.5683/SP2/1ZVWFV [5]
Data File 7	2017_AVERAGE_RAW (2017 folder)	.csv	Scholars Portal Dataverse, https://doi.org/10.5683/SP2/1ZVWFV [5]
Data File 8	2017_AVERAGE_STANDARDIZED (2017 folder)	.csv	Scholars Portal Dataverse, https://doi.org/10.5683/SP2/1ZVWFV [5]
Data File 9	2017_AVERAGE_SMOOTHED (2017 folder)	.csv	Scholars Portal Dataverse, https://doi.org/10.5683/SP2/1ZVWFV [5]
Data File 10	2017_AVERAGE_STANDARDIZED_SMOOTHED (2017 folder)	.csv	Scholars Portal Dataverse, https://doi.org/10.5683/SP2/1ZVWFV [5]
Data File 11	2018_FULL_ALLDATA (2018 folder)	.xlsx	Scholars Portal Dataverse, https://doi.org/10.5683/SP2/1ZVWFV [5]
Data File 12	2018_FULL_RAW (2018 folder)	.csv	Scholars Portal Dataverse, https://doi.org/10.5683/SP2/1ZVWFV [5]
Data File 13	2018_FULL_STANDARDIZED (2018 folder)	.csv	Scholars Portal Dataverse, https://doi.org/10.5683/SP2/1ZVWFV [5]
Data File 14	2018_FULL_SMOOTHED (2018 folder)	.csv	Scholars Portal Dataverse, https://doi.org/10.5683/SP2/1ZVWFV [5]
Data File 15	2018_FULL_STANDARDIZED_SMOOTHED (2018 folder)	.csv	Scholars Portal Dataverse, https://doi.org/10.5683/SP2/1ZVWFV [5]
Data File 16	2018_AVERAGE_ALLDATA (2018 folder)	.xlsx	Scholars Portal Dataverse, https://doi.org/10.5683/SP2/1ZVWFV [5]
Data File 17	2018_AVERAGE_RAW (2018 folder)	.csv	Scholars Portal Dataverse, https://doi.org/10.5683/SP2/1ZVWFV [5]
Data File 18	2018_AVERAGE_STANDARDIZED (2018 folder)	.csv	Scholars Portal Dataverse, https://doi.org/10.5683/SP2/1ZVWFV [5]
Data File 19	2018_AVERAGE_SMOOTHED (2018 folder)	.csv	Scholars Portal Dataverse, https://doi.org/10.5683/SP2/1ZVWFV [5]
Data File 20	2018_AVERAGE_STANDARDIZED_SMOOTHED (2018 folder)	.csv	Scholars Portal Dataverse, https://doi.org/10.5683/SP2/1ZVWFV [5]
Data File 21	README (Supplemental folder)	.txt	Scholars Portal Dataverse, https://doi.org/10.5683/SP2/1ZVWFV [5]
Data File 22	CODEBOOK (Supplemental folder)	.txt	Scholars Portal Dataverse, https://doi.org/10.5683/SP2/1ZVWFV [5]
Data File 23	GENETIC_MATERIALS (Supplemental folder)	.csv	Scholars Portal Dataverse, https://doi.org/10.5683/SP2/1ZVWFV [5]
Data File 24	2017_PHENOLOGY (Supplemental folder)	.csv	Scholars Portal Dataverse, https://doi.org/10.5683/SP2/1ZVWFV [5]
Data File 25	2018_PHENOLOGY (Supplemental folder)	.csv	Scholars Portal Dataverse, https://doi.org/10.5683/SP2/1ZVWFV [5]
Data File 26	SUPPLEMENTARY_IMAGES (Supplemental folder)	.pdf	Scholars Portal Dataverse, https://doi.org/10.5683/SP2/1ZVWFV [5]

## Data collection

### Experimental setup

The experiment was set up as a randomized complete block design with four replications, planted on May 12, 2017 and May 10, 2018 at the Elora Research Station (Elora, Ontario; 43° 38 ′ 27.0456" N, 80° 24′ 18.6948" W). Genotypes consisted of four extremely short-season hybrids, referred to as the Set 1 hybrids, and 12 short-season hybrids referred to as the Set 2 hybrids. The inbred line parents for two of the hybrids in Set 1 and for all Set 2 hybrids are publicly available and have been genotyped [6]. Set 1 hybrids were planted in a late planted experiment (May 29, 2017 and May 21, 2018) to sample spectral reflectance differences within a growing season. Environmental conditions such as air temperature, rainfall, wind speed and direction, and solar radiation were recorded at the Elora Weather Station, located on the Elora Research station. This information is publicly available for both 2017 [7] and 2018 [8].

### Data collection

Canopy-level hyperspectral measurements were taken using a ground based dual-channel reflectance spectrometer (Unispec-DC; PP Systems). This is a 243-channel sensor with a spectral range of 300.4–1101.8 nm, and a spectral resolution of 3.3 nm. Scans were calibrated using a spectralon tile (spectralon 12 × 12 inch calibrated white; ASD Inc) with 99.5% reflectance across the visual and near infrared spectrum. Sampling was done within 3 h of solar noon, typically after dew was gone from plants and on days without rain. Scans were taken starting 3 weeks post silking and continued every 2 to 4 days until physiological maturity, weather permitting.

## Limitations

Errors and missing data were dealt with in a defined manner. For the hyperspectral data collection, when machine errors occur, they are automatically given the value 9999 within the dataset, which were then replaced with a decimal and treated as missing data. Scans that were completely missed were populated with decimals and likewise treated as missing data. Zeros in the dataset were treated as true zeros, although it is possible that some of these are machine errors or the machine rounding down extremely small values rather than true zeros.

Although both years of data were planted at the Elora Research Station, due to crop rotation practices, the field which the trial was planted in changed year-to-year, and thus different soil environments may have been present. Weather played a large role on when sampling could occur, as conditions had to be dry as to not damage the machine, leading to different lengths of time between sampling dates.

## Data Availability

The datasets that were generated and described in this Data Note are publicly available and free to access at the Scholars Portal Dataverse repository, https://doi.org/10.5683/SP2/1ZVWFV [5].
